# Evaluierung und Optimierung des Neugeborenenscreenings mittels strukturierter Langzeitbeobachtung – am Beispiel der angeborenen Stoffwechselerkrankungen

**DOI:** 10.1007/s00103-023-03772-7

**Published:** 2023-10-10

**Authors:** Ulrike Mütze, Stefan Kölker

**Affiliations:** Sektion Neuropädiatrie und Stoffwechselmedizin, Zentrum für Kinder- und Jugendmedizin, Universitätsklinikum Heidelberg, Im Neuenheimer Feld 430, 69120 Heidelberg, Deutschland

**Keywords:** Sekundärprävention, Langzeitentwicklung, Beobachtungsstudien, Register, Outcome, Secondary prevention, Neonatal screening, Long-term outcome, Observational studies, Registry

## Abstract

Das Neugeborenenscreening (NGS) ist ein hoch erfolgreiches Programm der Sekundärprävention mit dem Ziel, schwere Folgeschäden von angeborenen, meist genetisch bedingten Erkrankungen durch möglichst frühe präsymptomatische Identifizierung zu verhindern. Bisherige Studien zeigen wichtige Errungenschaften von NGS-Programmen, decken aber auch eine Reihe von relevanten Schwächen auf. Dazu gehören der zumeist unvollständig verstandene natürliche Verlauf und die phänotypische Vielfalt seltener Krankheiten sowie die unzureichende Möglichkeit einer präzisen, frühen Vorhersage des individuellen Krankheitsschweregrads und damit die Unsicherheiten bei der Falldefinition, der Risikostratifizierung und der Behandlungsindikation.

Vor dem Hintergrund der rasanten Entwicklungen in den genetischen Hochdurchsatztechnologien und der damit verbundenen Möglichkeiten einer zukünftigen erheblichen Ausweitung der NGS-Programme erscheint es überfällig, die strukturierte Langzeitbeobachtung und die hierauf basierende Evaluation des langzeitlichen Gesundheitsnutzens für die im NGS identifizierten Menschen mit seltenen Krankheiten obligatorisch im NGS-Programm zu verankern. Der vorliegende Beitrag erläutert die Bedeutung der Langzeitbeobachtung für die Evaluation und die kontinuierliche Optimierung des NGS. Klinische Langzeitverläufe der im NGS identifizierten Menschen mit angeborenen Stoffwechselkrankheiten werden beispielhaft dargestellt.

## Abstract (en)

Newborn screening (NBS) is a highly successful secondary prevention program with the goal of preventing severe sequelae of congenital, mostly genetic, diseases by identifying them as early as possible, ideally in the pre-symptomatic period. Studies to date have shown the important achievements of NBS programs but also reveal a number of relevant weaknesses. These include the often incompletely understood natural history and phenotypic diversity of rare diseases as well as the inadequate ability to accurately predict individual disease severity at an early stage and thus the uncertainties in case definition, risk stratification, and treatment indication.

In light of the rapid developments in high-throughput genetic technologies and the associated opportunities for substantial future expansion of NBS programs, it seems overdue to make structured long-term follow-up and the subsequent evaluation of the long-term health benefits mandatory for individuals with rare diseases identified through NBS. This article explains the importance of long-term follow-up for the evaluation and continuous optimization of the screening. Long-term clinical outcomes of people with inherited metabolic diseases identified by NBS are presented as examples.

## Einleitung

Das Neugeborenenscreening (NGS) ist eine hoch erfolgreiche sekundäre Präventionsmaßnahme mit dem Ziel der Verhinderung schwerer Folgeschäden angeborener, meist genetisch bedingter Erkrankungen durch eine frühe, möglichst präsymptomatische Identifizierung, Diagnose und zeitnahe Einleitung einer Therapie. Seit Einführung des NGS auf Phenylketonurie (PKU) Ende der 1960er-Jahre wurde die Auswahl der Zielkrankheiten weltweit kontinuierlich erweitert [1–3]. Dies wurde insbesondere durch den Einsatz innovativer analytischer Hochdurchsatzverfahren wie der Tandem-Massenspektrometrie Ende der 1990er-Jahre befördert und führte zur Aufnahme von 10 weiteren angeborenen Stoffwechselkrankheiten in das „Erweiterte NGS“ ab 2005 [4]. In den letzten Jahren führten hochdurchsatzfähige molekulargenetische Verfahren, die für die Frühdiagnose von zystischer Fibrose [5], schweren kombinierten Immundefekten (SCID), Sichelzellkrankheit (SCD) und spinaler Muskelatrophie (SMA; [6, 7]) genutzt werden können, erneut zu Erweiterungen des nationalen NGS-Programms in Deutschland. Aktuell umfasst das deutsche Regel-NGS 19 Einzelkrankheiten: 13 angeborene Stoffwechselkrankheiten, 2 Endokrinopathien, zystische Fibrose, SCID, SCD und SMA (Tab. 1).

**Table 1:** 

Zielkrankheiten		Geschätzte Prävalenz in Deutschland	Vorhandensein von aktuellen Leitlinien (≤ 5 Jahre alt; AWMF), alternativ internationale evidenzbasierte Empfehlungen	
			Konfirmationsdiagnostik nach dem NGS	Therapie und Monitoring
**Angeborene Stoffwechselkrankheiten**				
**1**	Phenylketonurie (PKU)	1:10.000	AWMF-Leitlinie 027-021 [72]	AWMF-Leitlinie in Vorbereitung (027-002) Internationale Empfehlungen (Europa [73]; Frankreich [74])
**2**	Tyrosinämie Typ 1	1:135.000		AWMF-Leitlinie 27-003 [75]
**3**	Ahornsirupkrankheit (MSUD)	1:180.000		Keine
**4**	Isovalerianazidurie (IVA)	1:90.000		Keine
**5**	Glutarazidurie Typ 1 (GA1)	1:140.000		AWMF-Leitlinie 27-018 [76]
**6**	Mittelketten-Acyl-CoA-Dehydrogenase-Mangel (MCADD)	1:10.000		Keine
**7**	Langketten-Acyl-CoA-Dehydrogenase-Mangel (VLCADD)	1:80.000		Keine
**8**	Langketten-Hydroxyacyl-CoA-Dehydrogenase-Mangel/Mangel an mitochondrialem trifunktionellen Protein (LCHADD/mTFPD)	1:160.000		Keine
**9** **10** **11**	***Carnitinstoffwechselstörungen*** *Carnitin-Palmitoyltransferase-1-Mangel (CPT1)* *Carnitin-Palmitoyltransferase-2-Mangel (CPT2)* *Carnitin-Acylcarnitin-Translokase-Mangel (CACT)*	1:600.000		Keine
**12**	Biotinidase-Mangel	1:28.000		Keine
**13**	Klassische Galaktosämie	1:75.000		Internationale Empfehlung [77]
**Endokrinopathien**				
**14**	Adrenogenitales Syndrom	1:15.000	AWMF-Leitlinie 174-003 [78]	
**15**	Kongenitale Hypothyreose	1:3000	Internationale Empfehlung [79]	
**Weitere**				
**16**	Zystische Fibrose	1:4500	AWMF-Leitlinie 026-024 [80]	
**17**	Schwere kombinierte Immundefekte (SCID)	1:30.000	AWMF-Leitlinie in Vorbereitung (198-002)	
**18**	Sichelzellkrankheit (SCD)	1:5000	AWMF-Leitlinie 025-016 [81]	
**19**	Spinale Muskelatrophie (SMA)	1:7000	AWMF-Leitlinie 022-030 [82]	

*AWMF* Arbeitsgemeinschaft der Wissenschaftlichen Medizinischen Fachgesellschaften e. V. (www.awmf.org). Die Durchführung des nationalen NGS in Deutschland basiert für alle Zielkrankheiten auf der AWMF-Leitlinie 024-012 [71]

### Von der symptombezogenen Medizin zur sekundären Prävention – vom kranken Menschen zum asymptomatischen Neugeborenen

Im Kontrast zum traditionell symptombasierten Konzept der Medizin besteht die wesentliche Herausforderung aller Screening-Programme darin, asymptomatische Menschen mit einem hohen Risiko für das Auftreten einer bestimmten Erkrankung idealerweise bereits im präklinischen Zustand anhand von diagnostischen Biomarkern zu identifizieren, zwischen „physiologischen“ und „pathologischen“ Zuständen klar zu unterscheiden und eine verlässliche Prädiktion des individuellen Erkrankungsverlaufs vorzunehmen. Nur wenn diese Voraussetzungen erfüllt sind, ist die Behandlung einer bislang asymptomatischen Person – im NGS ist diese das asymptomatische Neugeborene – zu rechtfertigen.

Hierzu werden Screening-Untersuchungen, zu denen das NGS gehört, anhand der bereits 1968 von Wilson und Jungner formulierten und weltweit anerkannten Prinzipien geprüft [8]. Diese setzen eine hohe Spezifität und Sensitivität der Screening-Methoden als Hochdurchsatzanalyse, Kenntnisse der Prävalenz und des natürlichen Verlaufs der Erkrankungen, das Vorhandensein einer wirksamen Therapie und Vorteile einer frühen Diagnose voraus. In den einzelnen Ländern wird dann von den entsprechenden regulatorischen Stellen und Gremien über die Aufnahme von Erkrankungen in das NGS-Programm entschieden. In Deutschland entscheidet nach entsprechendem Antrag und Aufnahme eines Bewertungsverfahrens der Gemeinsame Bundesausschuss (G-BA) unter Anhörung stellungnahmeberechtigter Organisationen und der Bewertung des Nutzens durch das Institut für Qualität und Wirtschaftlichkeit im Gesundheitswesen (IQWiG; [9]).

Die unterschiedliche Interpretation und Umsetzung der Prinzipien von Wilson und Jungner führen europa- und weltweit zu erheblichen Unterschieden in der Auswahl der NGS-Zielkrankheiten [1–3]. Bislang fokussieren sich viele NGS-Studien auf die technische Machbarkeit und die diagnostische Prozessqualität neuer Screening-Verfahren [10, 11], während die klinische Langzeitentwicklung und der Gesundheitsnutzen früh diagnostizierter und behandelter Kinder und Jugendlicher selten und zumeist wenig systematisch untersucht wurden. Dadurch bedingt blieben der langfristige Nutzen des NGS und das Potenzial für dessen Optimierung weitgehend unbekannt. Mit dem Ziel, dass Screening-Untersuchungen in der Zusammenschau aller Aspekte (individuell, gesellschaftlich, ökonomisch etc.) mehr nutzen als schaden sollen [12], wurde in den letzten Jahren, auch im Angesicht der neuen genetischen Diagnostikmethoden, eine Erweiterung der Screening-Kriterien nach Wilson und Junger insbesondere um die Evaluation des Nutzens für die gescreente Population gefordert [13, 14].

Nach fast 20-jährigem Bestehen des „erweiterten NGS“ zeigen wir in der vorliegenden Arbeit die Bedeutung der Langzeitbeobachtungen für die Evaluation und kontinuierliche Optimierung des NGS-Programms auf. Klinische Langzeitverläufe der im NGS identifizierten Menschen mit angeborenen Stoffwechselkrankheiten werden beispielhaft dargestellt.

## Der Nutzen der Langzeitbeobachtung

Menschen mit PKU ermöglicht das NGS durch den frühzeitigen Start einer diätetischen Therapie bereits seit über 50 Jahren eine normale Entwicklung und ein Leben ohne körperliche und kognitive Beeinträchtigung im Kindes‑, Jugend- und Erwachsenenalter. Dieser Erfolg gilt als Richtwert für alle nachfolgend eingeführten Zielkrankheiten. Allerdings ist es meist nicht möglich, dass alle Daten, die zur Erfüllung der von Wilson und Jungner geforderten Kriterien [8] notwendig sind, vor der Einführung einer neuen NGS-Krankheit vorliegen. Dies gilt insbesondere auch für den Verlauf von erst durch das Screening identifizierten geringeren Krankheitsausprägungen [15, 16].

Daten zur Langzeitentwicklung nach „erweitertem NGS“ wurden und werden Screening-begleitend in aufwändigen dezentralen Studien erhoben. Erste kleinere Kohorten mit kürzeren Beobachtungszeiträumen erbrachten positive Hinweise [17–20]. Nach nunmehr über 20 Jahren Erfahrung mit dem erweiterten NGS belegen die strukturierte Nachuntersuchung der NGS-Kohorte für angeborene Stoffwechselkrankheiten in Südwestdeutschland (*N* = 306 Kinder, mittlere Beobachtungsdauer 6,2 Jahre [21]) sowie nationale Kohorten für Einzelkrankheiten, insbesondere für die Glutarazidurie Typ 1 (GA1; nationale Erhebung; *N* = 87; [22]) und die Isovalerianazidurie (IVA; *N* = 94; [16, 23]), den Gesundheitsnutzen und langfristig positiven Einfluss der frühen Diagnosestellung und Therapie auf die klinische und kognitive Entwicklung für Kinder mit diesen seltenen Krankheiten.

Aus der Vogelperspektive betrachtet erzielen gescreente Menschen mit angeborenen Stoffwechselkrankheiten hervorragende gesundheitliche Ergebnisse: Eine normale frühe Entwicklung und Intelligenz (95 %), einen symptomfreien Verlauf ohne bleibende krankheitsspezifische Symptome (76 %), eine geringe Mortalität [21] und ein weitgehend normales Wachstum und Gedeihen [24]. Das ist ein wichtiger Erfolg für Kinder, die ohne frühe Diagnose und Therapie schwere Entwicklungsstörungen und Einschränkungen hätten oder von einem vorzeitigen Tod bedroht wären. In der näheren Betrachtung finden sich jedoch krankheitsspezifische Einschränkungen, die den Optimierungsbedarf bei einer Vielzahl von Aspekten deutlich machen und den Nutzen der Langzeitbeobachtung im NGS-Programm unterstreichen. Diese werden im Folgenden ausgeführt.

## Optimierung der Prozesszeiten zur Vermeidung von Stoffwechselentgleisungen

Erweiterte NGS-Programme umfassen eine wachsende Zahl von angeborenen metabolischen Erkrankungen mit dem Risiko einer (neonatalen) Stoffwechselentgleisung. Eine kürzlich durchgeführte Studie zeigte, dass noch bevor das NGS-Ergebnis bekannt war, 14,7 % (28 von 191) der gescreenten Kinder, die eine Erkrankung mit einem Risiko für eine Stoffwechselentgleisung hatten, eine solche bereits im Neugeborenenalter erlitten (medianes Alter: 4 Tage; [21]). Eine metabolische Dekompensation in den ersten Lebenstagen war hierbei jedoch nicht zwangsläufig mit einer ungünstigen langfristigen Entwicklung betroffener Kinder verbunden [21]. Allgemein zeigten sich aber deutliche krankheitsspezifische Unterschiede hinsichtlich der Vermeidbarkeit von Stoffwechselentgleisungen im weiteren Leben durch das NGS: Bei einigen Krankheiten (u. a. IVA, Mittelketten-Acyl-CoA-Dehydrogenase-Mangel – MCADD) entgleisten nur eine geringe Anzahl gescreenter Kinder (< 15 %), während andere (u. a. Langketten-3-Hydroxyacyl-CoA-Dehydrogenase‑/Mangel an mitochondrialem trifunktionellen Protein – LCHAD/MTPD und Ahornsirupkrankheit – MSUD) trotz NGS mehrheitlich (> 60 %) Stoffwechselkrisen erlitten [21].

**Figure 1:**
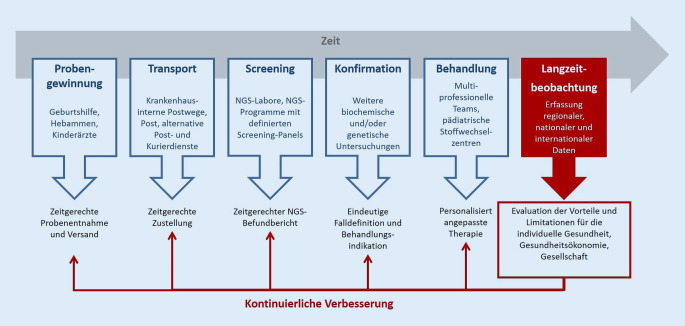

Da sich die erste Stoffwechselkrise überwiegend direkt nach der Geburt ereignete, ist der Zeitpunkt der Mitteilung des NGS-Ergebnisses von großer Relevanz, um Neugeborene vor dem Auftreten erster Symptome zu identifizieren. Damit dieses gelingen kann, zählt jeder Tag, beginnend mit der Probenentnahme über den Transport bis zur Fertigstellung des Befundes (Abb. 1). Eine kürzliche Untersuchung [21] konnte zeigen, dass in den letzten 15–20 Jahren das Alter bei Probenentnahme zwar gesenkt werden konnte, jedoch die Transportzeiten zunahmen und damit die in der Kinder-Richtlinie des G‑BA geforderte gesamte NGS-Prozesszeit von der Abnahme der Blutprobe bis zur Übermittlung des Befundes von 72 h [6] mittlerweile in mehr als 50 % der Fälle überschritten wird [21]. Hier besteht für den Erhalt der Qualität des NGS als auch im Hinblick auf eine potenziell zukünftige NGS-Erweiterung um Zielkrankheiten mit hoher neonataler Entgleisungsfrequenz [25] großer Handlungsbedarf, um einer Verlängerung der Prozesszeiten entgegenzuwirken. Trotz Optimierung dieser Abläufe werden jedoch einzelne, sehr frühe und dabei tödlich verlaufende neonatale Entgleisungen, wie z. B. bei MCADD [26], nicht vollständig verhindert werden können.

## Mögliche bleibende Symptome trotz Neugeborenenscreenings

Wie oben dargestellt entwickeln gescreente Kinder und Jugendliche mit angeborenen Stoffwechselkrankheiten nach früher Identifizierung im NGS und Einleitung einer spezifischen Therapie zumeist keine dauerhaften krankheitsspezifischen Symptome [21] und wachsen und gedeihen regelrecht [24]. Die Detailanalyse zeigt jedoch krankheitsspezifische Unterschiede: eine reduzierte kognitive Leistungsfähigkeit (MSUD [21], klassische IVA [16]; GA1 (Hochausscheider [27], Galaktosämie [21]), wiederholte Stoffwechselentgleisungen (MSUD; langkettige Fettsäurenoxidationsstörungen [21]) oder Abweichungen im Wachstum und Gedeihen [24]: Mit Ausnahme der PKU (Gewicht; [24, 28]) und GA1 (Kopfumfang; [29]) zeigen gescreente Kinder mit angeborenen Stoffwechselkrankheiten regelrechte Geburtsmaße [24].

Jedoch, wie bereits für die PKU gezeigt [28], können die Identifizierung durch das NGS und die anschließende frühzeitige (diätetische) Behandlung nicht vollständig vor Wachstumsveränderungen schützen. Für die metabolischen Erkrankungen des NGS zeigt die Längsschnittanalyse der anthropometrischen Maße bis zum Alter von 18 Jahren im Vergleich zur Referenzpopulation eine geringere Körpergröße (Standard Deviation Score: −0,5 SDS; *p* < 0,0001) und einen leicht geringeren Kopfumfang (−0,2 SDS; *p* = 0,0028) bei vergleichbarem Gewicht (0,1 SDS; *p* = 0,5097). Der Body-Mass-Index (BMI) lag demgemäß höher (0,4 SDS; *p* < 0,0001). Diese Ergebnisse waren besonders ausgeprägt, wenn diätetische Maßnahmen (protein-, langkettige Triglycerid- und Galaktose-arme Ernährung) erforderlich waren (*p* < 0,001; [24]).

## Eindeutige Falldefinitionen und Kriterien für die Therapieindikation werden benötigt

Der Schweregrad des sich entwickelnden klinischen Phänotyps eines Individuums mit einer genetisch bedingten Erkrankung, wie z. B. einer angeborenen Stoffwechselkrankheit, befindet sich auf einem kontinuierlichen Spektrum [30]. Eine präzise Vorhersage des individuellen Schweregrades vor dem Auftreten erster klinischer Symptome zu geben, ist deshalb eine große Herausforderung. Allerdings bedarf ein im NGS identifiziertes asymptomatisches Neugeborenes für die Entscheidung zur Therapie einer klaren Falldefinition und einer möglichst genauen Vorhersage des zukünftigen Schweregrads.

**Figure 2:**
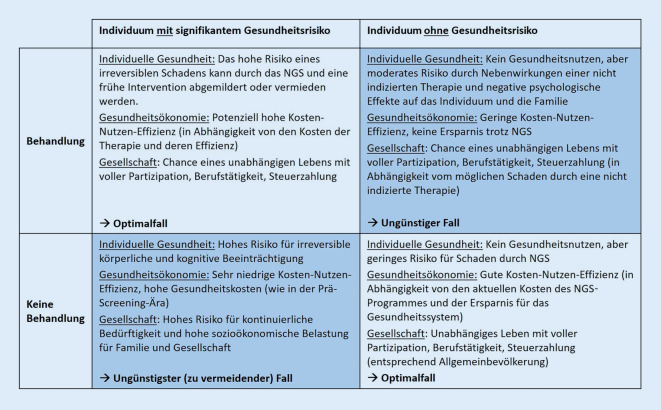

Mit der Einführung des NGS stieg die Geburtsprävalenz einiger Zielkrankheiten im Vergleich zum vorangegangenen Zeitraum an (Beispiele: MCADD [15, 31] und IVA [16, 32]). Dieser Unterschied erklärt sich zum einen aus der hohen diagnostischen Dunkelziffer vor der Einführung des NGS, zum anderen jedoch auch aus der zusätzlichen Identifizierung von Menschen mit bis dato unbekannten und möglicherweise benignen Verlaufsvarianten [16, 33]. Diese erweitern in den NGS-Kohorten das Spektrum des bekannten klassischen Phänotyps hin zu Manifestationen mit einem abgeschwächten Verlauf. Dies birgt das Risiko, dass der Gesundheitsnutzen von NGS-Programmen überschätzt wird, wenn die klinischen Schweregrade der NGS- und Prä-NGS-Kohorten deutlich voneinander abweichen. Zudem birgt die Identifizierung von Menschen mit benignen, nicht therapiebedürftigen Verlaufsvarianten die Gefahr, dass die Einleitung einer Therapie für diese Menschen keinen Zusatznutzen bringt, sondern sogar mit der Gefahr des Auftretens unerwünschter Wirkungen der nicht benötigten Therapie einhergehen kann (Abb. 2).

Große Anstrengungen müssen daher unternommen werden, eindeutige Falldefinitionen und Kriterien für die Therapieindikation vor Beginn des NGS festzulegen. Bei seltenen Krankheiten wie den angeborenen Stoffwechselkrankheiten des NGS kann dies in Pilotstudien an nationalen oder internationalen Kohorten herausgearbeitet werden. Die Evaluation der Langzeitverläufe ermöglicht hierbei die Erarbeitung exakter Falldefinitionen, die Identifizierung prädiktiver Biomarker und in der Folge einer stratifizierten Therapie [16, 23, 34, 35]. So konnte gezeigt werden, dass sich Kinder mit einer abgeschwächten Ausprägung der IVA, die sich bei 80 % der im Screening identifizierten Kinder findet, therapieunabhängig exzellent entwickeln [16], und eine neue Studie zeigt frühe Möglichkeiten der Prädiktion des klinischen Schweregrades für diese Kohorte auf [23]. Diese Erkenntnis kann viele Kinder und Familien vor Übertherapie und damit vor Schaden infolge des NGS bewahren.

## Ermöglichung evidenzbasierter Leitlinien auf der Grundlage von Langzeitbeobachtung

**Figure 3:**
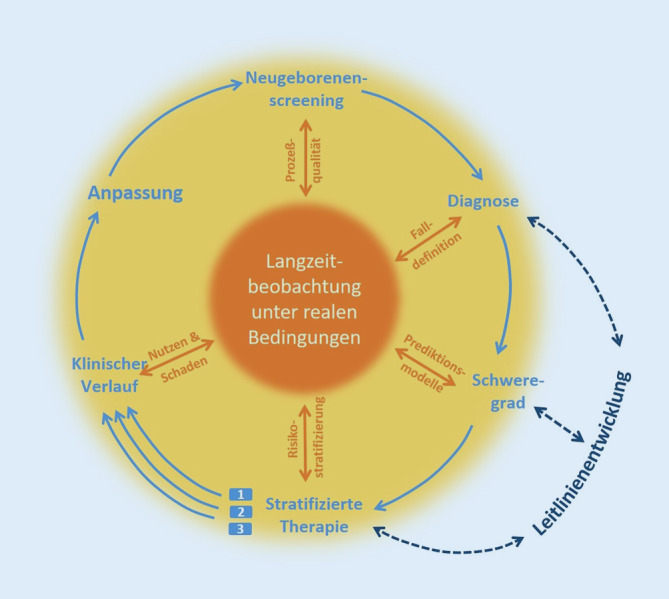

Langzeitbeobachtungsstudien ermöglichen auch die Evaluation der angewandten Therapien und damit die Entwicklung von Leitlinien (Abb. 3). Ein erfolgreiches Beispiel hierfür ist die GA1. Die prognostisch entscheidende Krankheitsmanifestation ist das Auftreten einer irreversiblen dystonen Bewegungsstörung als Folge einer Schädigung des Striatums, das Teil der Basalganglien im Gehirn ist. Dieses Ereignis tritt akut oder schleichend beim bis dahin asymptomatischen Säugling oder Kleinkind auf [36, 37]. Eine erst nach Auftreten der Symptome begonnene Behandlung führt nicht zu einer Verbesserung des klinischen Outcomes [38]. Daher wurde die GA1 in der Hoffnung, dass ein präsymptomatischer Behandlungsbeginn das Auftreten von Symptomen verhindern könnte, in den späten 1990er-Jahren in erste NGS-Pilotstudien und nationale NGS-Programme aufgenommen, die diesen Nutzen nachfolgend nachweisen konnten [36, 37]. Dieser erste Erfolg war der Ausgangspunkt für die Entwicklung einer internationalen Leitlinie [39] und einer Reihe von Langzeitbeobachtungsstudien mit GA1-Betroffenen. Diese zeigten, dass eine Notfallbehandlung während kataboler Episoden die wirksamste Maßnahme zur Vermeidung einer (akut auftretenden) Dystonie ist [40–42], eine Carnitin-Supplementierung die Sterblichkeitsrate senkt, Riboflavin aber keine nachweisbare positive Wirkung zeigt [36] und dass beide GA1-Phänotypen (sog. Hoch- und Niedrigausscheider) unbehandelt ein hohes Risiko für das Auftreten striataler Schäden aufweisen [36]. Schließlich gelang zudem der wichtige Nachweis, dass eine balancierte, Lysin-arme Diät unter Supplementation mit Lysin-freien, Arginin- und Tryptophan-angereicherten Aminosäurenpräparaten gegenüber einer proteinarmen Diät überlegen ist [40, 43]. Diese Ergebnisse führten zu kontinuierlichen Erweiterungen und Verbesserungen einer S3-Leitlinie [44–46], die mittlerweile in der vierten Fassung vorliegt (AWMF-Registernummer 027-018).

Nach fast 20 Jahren klinischer Forschung liegen somit solide Belege dafür vor, dass GA1 eine behandelbare Krankheit ist und dass ein positives neurologisches Outcome neben der Frühdiagnose mittels NGS wesentlich von einer hohen Therapiequalität und deren Aufrechterhaltung durch ein multiprofessionelles Team abhängt [22, 27, 40, 42, 47]. Zudem zeigen die fortlaufenden Beobachtungsstudien weitere Aspekte der Krankheit, wie chronische Nierenerkrankungen [22], fortschreitende Veränderungen der weißen Substanz [48] und einen leicht verringerten IQ bei Kindern und Jugendlichen der Hochausscheider-Gruppe [27]. Weitere Forschung ist erforderlich, um wirksame Therapien zu finden, die diese Veränderungen aufhalten können.

## Spezialisierte lebenslange Versorgung

Durch das NGS identifizierte und frühzeitig behandelte Menschen erreichen in aller Regel das Erwachsenenalter und haben eine gute Lebenserwartung. Hieraus ergibt sich ein wachsender Bedarf an spezialisierter lebenslanger Versorgung und eine Anpassung der Versorgung an altersbezogene Erfordernisse und Besonderheiten, inklusive einer kompetenten Betreuung bei Schwangerschaften. Während allerdings fast alle Universitätskliniken spezialisierte pädiatrische Ambulanzen vorhalten, sind Ambulanzen für Erwachsene mit angeborenen Stoffwechselkrankheiten derzeit noch nicht flächendeckend vorhanden und etablierte Transitionsmodelle [49, 50] selten. Dabei ist gerade für das den Großteil des Lebens ausmachende Erwachsenenalter eine spezialisierte und regelmäßig evaluierte, evidenzbasierte Therapie mit Anpassung an spezielle Bedürfnisse und Anforderungen [51–54] notwendig.

## Zukünftige Erweiterung des Neugeborenenscreenings

Seit dem Start vor über 50 Jahren ist das NGS ein dynamisches Programm, das parallel zu technischen Innovationen weiterentwickelt wurde. Jedoch ist die Weiterentwicklung des Programms nicht allein an technische Bedingungen gekoppelt, sondern erfordert zudem die Erfüllung von Voraussetzungen, die von Wilson und Jungner in den 1960er-Jahren für die WHO erarbeitet wurden [8]. Diese Screening-Kriterien für seltene Krankheiten vollständig zu erfüllen, ist vor einer Aufnahme in das NGS häufig nicht sicher gewährleistet, wodurch die oben dargestellten Unsicherheiten bezüglich der Falldefinition und Therapieindikationsstellung resultieren [16]. Daher sind Pilotstudien vor Aufnahme in das NGS sowie eine anschließende begleitende Evaluierung des Gesundheitsnutzens durch Langzeitbeobachtungsstudien unerlässlich, um die vorhandenen Wissenslücken kontinuierlich zu schließen und das NGS-Programm iterativ zu optimieren (Abb. 1 und Abb. 3).

Ein Beispiel ist der neonatale Vitamin‑B_12_-Mangel. NGS-Pilotstudien konnten zeigen, dass ein NGS hier technisch möglich ist [55–58]. Eine Nachbeobachtungsstudie ergab, dass sich alle Kinder mit neonatalem Vitamin‑B_12_-Mangel, die durch das NGS identifiziert wurden, nach einer ausschließlich oralen Therapie bis in das zweite Lebensjahr hinein altersentsprechend entwickelten [59]. Zudem profitierten viele Mütter, deren eigener Vitamin‑B_12_-Mangel bis dahin unerkannt geblieben war und die Ursache für den Vitamin‑B_12_-Mangel ihrer neugeborenen Kinder bildete, ebenfalls vom NGS und der hierdurch ermöglichten weiteren diagnostischen Abklärung und Therapie [60]. Diese Studien haben dazu geführt, dass die Aufnahme des neonatalen Vitamin‑B_12_-Mangels in das Regel-NGS zusammen mit weiteren, im Rahmen der Abklärung ebenfalls identifizierbaren Krankheiten (Homozystinurie, Methylmalonazidurie, Propionazidämie; [61]) aktuell geprüft wird [62]. Die präventive Bedeutung eines solchen NGS für die Entwicklung eines Vitamin‑B_12_-Mangels im späteren Säuglingsalter war noch nicht abschließend geklärt [63]. Eine aktuelle Erhebung zum infantilen Vitamin‑B_12_-Mangel in Kooperation mit der Erhebungseinheit für Seltene Pädiatrische Erkrankungen in Deutschland (ESPED)^1^ konnte nun aber darstellen, dass das Risko für einen symptomatischen infantilen Vitamin‑B_12_-Mangel bei Vorhandensein eines Pilotscreenings auf neonatalen Vitamin B_12_-Mangel um den Faktor 4 geringer ist, verglichen mit Kindern ohne Zugang zu einem solchen Screening [64, 65].

## Gesellschaftliche und gesundheitsökonomische Aspekte des Neugeborenenscreenings

Neben dem individuellen gesundheitlichen Nutzen für die Identifizierten sollten NGS-Programme für die untersuchte Bevölkerung, die Gesellschaft und die Gesundheitssysteme machbar und angemessen sein [8]. Die Kosten der Therapie für im NGS Identifizierte sowie der Einrichtung und Aufrechterhaltung der notwendigen Infrastrukturen müssen mit den Kosten der Therapien nicht gescreenter erkrankter Menschen verglichen und mit den weiteren Kosten oder Einsparungen für die Gesellschaft korreliert werden ([66–68]; Abb. 2). Hierfür müssen soziodemografische Merkmale wie Bildung, Beschäftigung, Elternschaft in erwachsenen Kohorten [49, 50] erfasst und ausgewertet werden. Darüber hinaus sollten unterschiedliche Behandlungsoptionen bei wirtschaftlichen Bewertungen berücksichtigt werden. So konnte für die USA gezeigt werden, dass das NGS auf PKU mit einer phenylalaninarmen Diät gesundheitsökonomisch kosteneffektiv war, während sich keine Kosteneffektivität für die Kofaktortherapie zeigte [69]. Analog dazu wird die Einführung höchstpreisiger Gentherapien und anderer neuartiger Therapien für (künftige) NGS-Zielkrankheiten eine sorgfältige Bewertung der wirtschaftlichen Folgen aus gesellschaftlicher Sicht und die Etablierung innovativer Preismodelle erfordern, insbesondere wenn bereits etablierte Therapien zur Verfügung stehen.

## Langzeitbeobachtung sollte in Zukunft das Neugeborenenscreening begleiten

Gray und Kollegen formulierten die wichtige Erkenntnis, dass Screening-Programme positive und negative Auswirkungen haben können und dass bisher nur einige von ihnen mehr positive als negative Effekte zu annehmbaren Kosten haben [12]. Es ist wichtig, dies anzuerkennen, es bei der Planung einer Ausweitung von NGS-Programmen zu berücksichtigen und unermüdlich an der Verbesserung der Qualität der bereits bestehenden Programme zu arbeiten. Langzeitbeobachtungstudien sind dafür elementare Werkzeuge (Abb. 3): Sie helfen, (1) den natürlichen Verlauf und die phänotypische Vielfalt seltener Krankheiten besser zu verstehen, (2) individuelle Krankheitsverläufe frühzeitig und präzise vorherzusagen, (3) die Unsicherheit bei der Falldefinition, der Risikostratifizierung und der Behandlungsindikation zu verringern, (4) den individuellen gesundheitlichen Nutzen sowie den wirtschaftlichen und gesellschaftlichen Nutzen von NGS-Programmen zu bewerten und (5) die kontinuierliche Anpassung und Verbesserung dieser zu ermöglichen. Begleitet wird dieser Prozess durch eine parallele Leitlinienentwicklung und stetige Aktualisierung, die für die im NGS identifizierten Menschen eine flächendeckende und bestmögliche Therapie bei sehr seltenen Erkrankungen erlauben. Allerdings sind diese Möglichkeiten bisher keineswegs ausgeschöpft, was sich unter anderem an dem noch unvollständigen Vorliegen evidenzbasierter Leitlinien für aktuelle NGS-Zielkrankheiten zeigt (Tab. 1). Dies lässt sich u. a. damit begründen, dass Langzeitbeobachtungsstudien und Leitlinienentwicklungen sehr zeit-, personal- und kostenintensiv sind.

Ohne eine kontinuierliche Evaluation im Rahmen von Langzeitbeobachtungsstudien mit gescreenten Menschen erfüllt das bestehende NGS in Deutschland bislang formal nicht die Kriterien eines Public Health Programmes, das regelmäßig evaluiert und optimiert werden sollte. Angesichts der oben dargestellten Erfolge (hinsichtlich der Versorgung und Therapie der gescreenten Menschen) sowie der Herausforderungen durch aktuelle und zukünftige Erweiterungen des NGS mit zum Teil neuen genetischen, kostenintensiven Therapien ist ein Verzicht auf die institutionelle Etablierung von Langzeitbeobachtungsstudien und -registern nicht mehr vertretbar.

Daher erscheint es überfällig, die Screening-Kriterien von Wilson und Jungner [8] zu revidieren und die strukturierte Evaluation des langfristigen Gesundheitsnutzens des NGS für Menschen mit seltenen Krankheiten, die im NGS identifiziert wurden, strukturell fest zu verankern [14]. Der Aufbau eines institutionell unterstützten NGS-Registers unter Einhaltung ethisch-rechtlicher Anforderungen, der allgemeinen Datenschutzverordnung^2^ und der FAIR-Datengrundsätze^3^ sowie der Integration der Perspektiven von Patienten, Familien und der Gesellschaft erscheint hierzu unerlässlich. Eine Vernetzung im europäischen und internationalen Kontext unter Einbeziehung europäischer Referenznetzwerke und deren Patientenregister ist zudem wünschenswert [70], um die bestehende Datenfragmentierung, parallele Aktivitäten und die Forschung an kleinen Stichproben und andere Einschränkungen zu überwinden.

Zusammenfassend belegen Langzeitentwicklungsstudien den großen Erfolg des NGS. Für den Erhalt und die stete Optimierung des NGS als umfassendes Programm ermöglichen Evaluationen der Langzeitentwicklung eine kontinuierliche Überprüfung, Anpassung und Optimierung des Screenings und der Therapie: Für ein bestmögliches Outcome der identifizierten Menschen und den größten Nutzen für die screenende und gescreente Gesellschaft.

## Footnotes

1 ESPED, Universität Mainz; https://www.unimedizin-mainz.de/esped/home/home.html (Zugriffsdatum 21.07.2023).

2 GDPR (General Data Protection Regulation), https://gdpr.eu (Zugriffsdatum 21.07.2023).

3 FAIR: Findable, Accessible, Interoperable, Reusable, s. https://www.go-fair.org/fair-principles/ (Zugriffsdatum 21.07.2023).
